# Escaping mTOR inhibition for cancer therapy: Tumor suppressor functions of mTOR

**DOI:** 10.1080/23723556.2017.1297284

**Published:** 2017-03-03

**Authors:** Victor H. Villar, Tra Ly Nguyen, Silvia Terés, Clément Bodineau, Raúl V. Durán

**Affiliations:** a Institut Européen de Chimie et Biologie, Pessac, France; b Institut Bergonié, ACTION Unit U1218 INSERM, Bordeaux, France; c University of Bordeaux, Bordeaux, France

**Keywords:** α-ketoglutarate, autophagy, cancer metabolism, glutamoptosis, mTORC1, rapamycin

## Abstract

A master promoter of cell growth, mammalian target of rapamycin (mTOR) is upregulated in a large percentage of cancer cells. Still, targeting mTOR using rapamycin has a limited outcome in patients. Our recent results highlight the additional role of mTOR as a tumor suppressor, explaining these modest results in the clinic.

The serine/threonine kinase mammalian target of rapamycin (mTOR) is a central regulator of mammalian cell growth. mTOR forms 2 complexes, termed mTOR complex 1 (mTORC1) and mTOR complex 2 (mTORC2). While both complexes are stimulated by growth factors, only mTORC1 can be activated by amino acids (^1^Cell Res). Particularly, the catabolism of glutamine (glutaminolysis), which yields α-ketoglutarate (αKG), activates the lysosomal translocation and subsequent activation of mTORC1 (^2^Mol Cell). Recently, our work revealed an unexpected mechanism by which the unbalanced activation of glutaminolysis in the absence of other amino acids induces a particular type of mTORC1-dependent cell death that we are naming “glutamoptosis” (^3^Nat Comm). During glutamoptosis, abnormally high levels of glutaminolytic αKG during nutrient restriction activates mTORC1, which in turn inhibits autophagy (^4^Autophagy). The inhibition of autophagy during glutamoptosis results in the accumulation of the autophagic protein sequestosome 1 (SQSTM1/p62), as SQSTM1/p62 is degraded during autophagy (^5^Cell). Our results showed that the increasing levels of SQSTM1/p62 in these restrictive conditions induce its interaction with caspase 8 to trigger apoptosis. The inhibition of mTORC1 reactivates autophagy and decreases SQSTM1/p62 levels, abrogating the induction of apoptosis by glutaminolysis. Thus, surprisingly, the inhibition of mTORC1 prevents glutamoptosis-mediated cell death, representing a tumor suppressor function of mTORC1 during nutritional imbalance.

For a long time, mTORC1 is known to be hyperactive in a large variety of different types of human cancer (^6^Cell). Therefore, this pathway has been considered as a major target for cancer therapy. However, for unclear reasons, the inhibition of mTORC1 as a therapeutic strategy has only modestly improved the outcome of patients (^7^N Engl J Med). Several reasons have been invoked to explain this lack of success in the use of rapamycin and analogues (rapalogues) in the clinic. The most accepted reason is the existence of a negative feedback loop downstream of mTORC1 which, upon its inhibition, upregulates the phosphoinositide 3-kinase (PI3K) signaling (^8^Curr Biol). The upregulation of the PI3K pathway would result in a deleterious effect of rapamycin treatment, as it promotes cancer growth, including the activation of mTORC2. To overcome this issue, dual inhibitors targeting both mTOR complexes have been designed, although it is unclear that they can actually improve the outcome of rapamycin in patients.

Our recent results suggest that additional fundamental reasons might explain the capacity of cancer cells to escape rapamycin treatment. As explained above, the inhibition of mTORC1 or the dual inhibition of both mTORC1 and mTORC2 during nutritional imbalance prevents glutamoptosis and promotes cell survival. Considering the restrictive nature of tumors (particularly solid tumors) due to their abnormal vasculature, and their general avidity to consume glutamine, tumors might constitute favorable microenvironments to induce glutamoptosis. In these conditions, the inhibition of mTORC1 during cancer therapy would prevent tumor growth, but at the same time would provide with an opportunity to cancer cells to avoid cell death. In other words, rapamycin treatment will result in a merely cytostatic effect, sustaining the survival of tumor cells. Upon treatment discontinuation, tumors will resume their growth. As a result, cancer progression will be only delayed during the period of rapamycin treatment or until the acquisition of rapamycin resistance mechanism by the cytostatic tumor cell.

These tumor suppressor functions of mTORC1 highlight the complexity of the action mechanisms of central cell growth regulators, such as mTOR, and how microenvironmental cues influence their function (Fig. 1). The assumption that the inhibition of these cell growth regulators will inevitably result in the arrest of tumor growth seems to be a too simplistic view of the complex mechanism of cell growth control. Further, it confirms that strategies to beat cancer based on targeted monotherapies, at least in the case of mTOR, will probably require further reconsideration to mitigate those adverse consequences. In the case of glutamoptosis, our results indicated that the re-stimulation of autophagy mediates the pre-survival effect of rapamycin. Thus, it can be envisioned that autophagy inhibition could certainly improve the outcome of rapamycin treatment by re-activating glutamoptosis. Indeed, treatments targeting both mTOR signaling and autophagy have been previously proposed and are already under clinical evaluation (^9^Autophagy). The lack of efficient and specific inhibitors of autophagy is a major limitation for the implementation of this strategy. The central role played by the autophagic protein SQSTM1/p62 during glutamoptosis and its close connection with mTORC1 suggest that SQSTM1/p62 upregulation might be a key element to overcome rapamycin-mediated cell survival. Our results already indicate that SQSTM1/p62 upregulation can indeed induce cell death even in rapamycin-treated cells.

**Figure 1.**
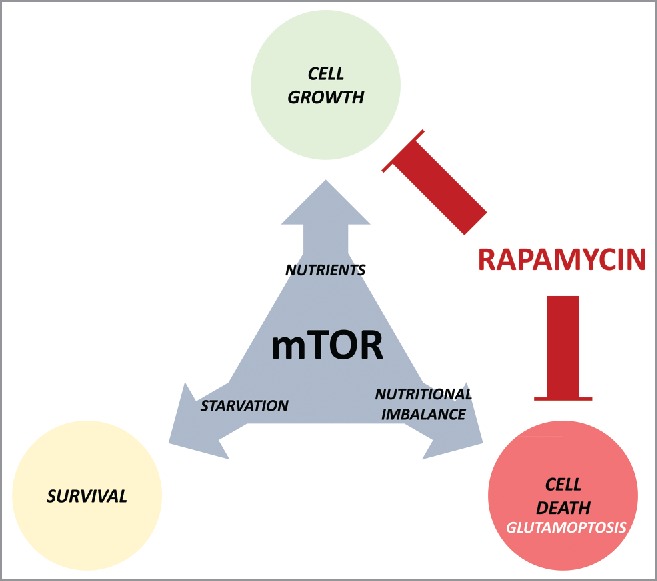
The many faces of the mammalian target of rapamycin (mTOR). mTOR promotes cell growth during nutrient availability, and its inactivation allows cell survival in nutrient-restrictive conditions. However, its anomalous activation induces cell death (glutamoptosis). Rapamycin treatment blocks cell growth, but at the same time guarantees cell survival during nutritional imbalance, a detrimental effect for cancer therapy.

Finally, our results open a new opportunity to explore the translational involvement of glutamoptosis for cancer therapy. In other words, can we induce glutamoptosis in tumors to specifically kill cancer cells? Microenvironments with a high concentration glutamine, or tumor types with a particular avidity for glutamine, might be particularly sensitive to glutamoptosis. Simulating glutaminolysis by artificially increasing the intracellular levels of αKG is known to induce tumor cell death *in vivo* (^10^Oncogene), although the involvement of mTORC1 and SQSTM1/p62 in this phenotype remains elusive.

In conclusion, the tumor suppressor function of mTORC1 during nutritional imbalance points at the necessity of finding alternatives to improve the outcome of mTORC1 inhibition in the clinics and questions the pertinence of the use of rapamycin as monotherapy in cancer patients.
